# Prophylactic ECMO Support during Elective Coronary Percutaneous Interventions in High-Risk Patients: A Single-Center Experience

**DOI:** 10.1155/2023/5332038

**Published:** 2023-02-04

**Authors:** Claudiu Ungureanu, Marc Blaimont, Hugues Trine, Pierre Henin, Romain Courcelle, Yves Laurent, Patrick Van Ruyssevelt, Caroline Lepièce, Vincent Huberlant

**Affiliations:** ^1^Cardiovascular Department, Jolimont Hospital, La Louvière, Belgium; ^2^Intensive Care Department, Jolimont Hospital, La Louvière, Belgium; ^3^Cardiothoracic Surgery Department, Jolimont Hospital, La Louvière, Belgium

## Abstract

**Introduction:**

Evidence regarding the impact of prophylactic implantation of venoarterial extracorporeal membrane oxygenation (VA-ECMO) for elective high-risk percutaneous coronary intervention (PCI) is limited. The purpose of this paper is to evaluate the outcome during index hospitalization and 3 years after interventions.

**Methods:**

This is an observational retrospective study including all patients undergoing elective, high-risk PCI and receiving VA-ECMO for cardiopulmonary support. Primary endpoints were in-hospital and 3- year major adverse cardiovascular and cerebrovascular event (MACCE) rates. Secondary endpoints were vascular complications, bleeding, and procedural success.

**Results:**

Nine patients were included in total. All patients were considered inoperable by the local heart team, and 1 patient had a previous coronary artery bypass graft (CABG). All patients were hospitalized for an acute heart failure episode 30 days before the index procedure. Severe left ventricular dysfunction was present in 8 patients. The main target vessel was the left main coronary artery in 5 cases. Complex PCI techniques were used: bifurcations with 2 stents in 8 patients, rotational atherectomy was performed in 3, and coronary lithoplasty in 1 case. PCI was successful in all of the patients with revascularization of all target and additional lesions. Eight of the 9 patients survived for at least 30 days after the procedure, and 7 patients survived for 3 years after the procedure. Regarding the complication rate, 2 patients suffered from limb ischemia and were treated by an antegrade perfusion, 1 patient had a femoral perforation that needed surgical repair, 6 patients had a hematoma, 5 patients had a significant drop in hemoglobin of more than 2 g/dl and received blood transfusions, 2 patients were treated for septicemia, and 2 patients needed hemodialysis.

**Conclusions:**

Prophylactic use of VA-ECMO in elective patients is an acceptable strategy for revascularization by high-risk coronary percutaneous interventions with good long-term outcomes for patients considered inoperable when a clear clinical benefit is expected. Regarding the potential risk of complications due to a VA-ECMO system, the selection of candidates in our series was based on a multiparameter analysis. The two main triggers in favor of prophylactic VA-ECMO in our studies were the presence of a recent heart failure episode and the high probability of periprocedural prolonged impairment of the coronary flow through the major epicardial artery.

## 1. Introduction

In the last few years, the number of patients with multiple comorbidities and previous cardiothoracic surgery that are not suitable for surgical revascularization has increased [1–4].

Until recently, treatment for these patients was either conservative or high-risk PCI (HR-PCI) with a high chance of periprocedural mortality [2].

As a result, percutaneous coronary intervention (PCI) has become more complex and most frequently performed in patients with challenging coronary lesions [5].

HR-PCI is performed usually in a population with one or more specific characteristics related to either patients (increased age, CKD, COPD, HVD, HF, MI, PAD, and TIA or stroke), lesions (stenosis involving the LM, last patent conduits, presence of a severe multivessel coronary artery disease, CTOs, and heavily calcified lesions), or the clinical presentation (hemodynamic status, left ventricular function, and presence or risk of electrical instability) [6, 7].

HR-PCI often induces transient myocardial ischemia that can lead to hemodynamic instability or a complete collapse in the case of complications; therefore, mechanical support seems to be an interesting theoretical solution [3, 4].

In the past, HR-PCI was performed using an intra-aortic balloon pump (IABP), but previous studies failed to show a clear benefit of this approach [8].

More recently, HR-PCI under mechanical support, especially VA-ECMO and the Impella device (Abiomed, Aachen, Germany), was feasible in this group of high-risk patients [9–13].

VA-ECMO is a form of temporary mechanical circulatory support and simultaneous extracorporeal gas exchange that is most commonly used in patients with cardiac arrest and cardiogenic shock with MI and heart and respiratory failure, allowing complete support by ensuring continuous systemic perfusion and oxygenation [9].

The current study aims at describing patient characteristics, procedural findings, and outcomes in the use of VA-ECMO for mechanical circulatory support in elective HR-PCI.

## 2. Methods

We performed a single-center, observational, retrospective study, including all patients that underwent prophylactic VA-ECMO for elective HR-PCI between May 2017 and January 2022. The mean number of ECMO implantation is 30 per year, and the program started 25 years ago.

Elective prophylactic VA-ECMO use was defined as the absence of any pharmacological or other mechanical support at the moment of the procedure.

All cases were discussed collectively by a heart team comprising one clinical cardiologist, two intensive care physicians, an anesthesiologist, a cardiac surgeon, and an interventional cardiologist. For all patients, surgical revascularization was excluded after the heart team evaluation due to high procedural risk.

Primary endpoints were in-hospital and 3-year major adverse cardiovascular and cerebrovascular event (MACCE) rates. Secondary endpoints were vascular complications, bleeding, and procedural success.

All patients were analyzed for age, gender, and previous medical history including diabetes, hypertension, peripheral artery disease, tobacco use, renal function, clinical presentation, angiographic, and transthoracic echocardiographic features. SYNTAX scores I and II were calculated in order to assess coronary anatomy using the online calculator.

The MACCE rate was defined as a composite of death, MI, TIA or stroke, and urgent target vessel revascularization (TVR). Periprocedural MI was defined using the Society for Cardiovascular Angiography and Interventions (SCAI) criteria, i.e., an increase in CK-MB of ≥10x the upper limit of normal within the first 72 hours of the procedure. MI after the periprocedural period was defined based on the third universal definition of MI. Bleeding events were evaluated according to the Bleeding Academic Research Consortium (BARC) classification. Angiographic success was defined as a final diameter stenosis of <30% as assessed by visual inspection with a final Thrombolysis in Myocardial Infarction (TIMI) flow grade of 3. eGFR <60 (stage 3) was considered impaired renal function.

Left ventricular function was defined as good (>55%), mildly impaired (45–55%), moderately impaired (35–45%), or severely impaired (<35%) [17]. Hemodynamic instability was defined as the need for at least one inotrope to maintain a mean arterial pressure >65 mmHg, while electrical instability was the recurrence of sustained ventricular arrhythmias in the last 24 h. [14–17]

## 3. VA-ECMO Procedure

The VA-ECMO system comprises a centrifugal pump, a membrane oxygenator, a heat exchanger, and bypass cannulas. Femoral vascular access (percutaneous or with surgical isolation) was performed under echography guidance. For the VA- ECMO implantation, a 22 F venous cannula was positioned in the right atrium under fluoroscopy guidance and an 18–20 Fr high-flow femoral arterial cannula was used.

After obtaining retrograde common femoral artery and anterograde superficial femoral access, a series of progressive dilations over an extrastiff 0.035″ guidewire were performed to allow the placement of the VA-ECMO cannula. The venous cannula was placed with a similar method using a series of dilators over an extrastiff wire.

The VA- ECMO cannulas were removed after the procedure by vascular surgery.

The mean VA-ECMO flow was maintained at about 3-4 L/min. The gas supplied to the oxygenator was adjusted to achieve a target oxygen saturation (SpO_2_) of at least 85% mmHg and normocapnia upon lung-protective ventilation. On VA-ECMO, the flow and gas supply rates were adjusted using blood gas examinations. At the end of the procedure, the pump flow rates were decreased to a value at which the patient was vasopressor-free (a minimum of 1 mL/kg/min was required in order to avoid clotting of the system) before VA-ECMO removal.

### 3.1. PCI Procedure

Second vascular access (conventional, distal radial, or femoral) was obtained for the intervention. All patients were pretreated at a loading dose of 600 mg of clopidogrel and 100 mg aspirin i.v. Heparin was given to achieve and maintain an activated clotting time (ACT) of >300 seconds; ACTs were monitored every 20 minutes. All PCIs were performed with 6-7 Fr guiding catheters. The PCI technique was performed at the discretion of the operator. Second-generationeverolimus-eluting stents were used in all patients.

## 4. Results

### 4.1. Baseline Clinical Characterstics

A total of 9 male patients were included and underwent high-risk PCI under prophylactic VA-ECMO support in a single Belgian center. The clinical characteristics are described in (Table 1).

Seven patients had ACS recorded within 30 days before the index procedure. All the patients had a recent acute heart failure episode (pulmonary edema, congestive heart failure, or cardiogenic shock before being transferred to our tertiary care hospital). The patients were stable without any type of support at the time of the procedure.

### 4.2. Baseline Characteristics (Cardiac History, Anatomy, and Risk Scores)

The main target vessel was the left stem coronary artery in 5 cases. The target vessel was a CTO in 1 patient. Median SYNTAX score I was 28.4 (12.4–18.9) (Table 2).

Severe left ventricular dysfunction was present in 8 patients. One patient had mild LV dysfunction but was admitted with pulmonary edema and in stage B cardiogenic shock with complex coronary disease during NSTEMI.

Only two patients were not completely revascularized because of the proof of no myocardial viability.

### 4.3. PCI Characteristics

The vascular access to PCI was the distal radial artery in 2 cases and the proximal radial artery in 6 cases, using 7 Fr guiding catheters for all the patients. Three patients had multivessel angioplasty (Table 3).

Complex PCI techniques were used: bifurcations with 2 stents in 8 patients by multiple approaches: the DK-crush technique in 2 patients, nanocrush in 2 cases, and TAP in 4 cases.

Rotational atherectomy was performed in 3 patients and coronary lithoplasty in 1 case.

### 4.4. Procedural Characteristics

Mechanical ventilation was used for all patients. The cardiovascular surgeon performed the cannulation in 7 patients and the intensive care physicians in 2 others.

A femoral-femoral approach was needed in all patients. In 7 patients, the arterial femoral cannula was introduced surgically in a Dacron tube grafted on the femoral artery. This technique is used to avoid the need for reperfusion of the superficial femoral artery. The 2 other patients received an antegrade perfusion of the leg during the procedure due to percutaneous cannulation of the femoral artery.

After the procedure, all patients were decannulated in the operating theatre by the cardiothoracic surgeon.

### 4.5. Outcome

PCI was successful in all of the patients with revascularization of all target and additional lesions. Eight patients survived for 30 days after the procedure, and 7 survived for 3 years after the procedure (Table 4).

One patient died during hospitalization due to hemorrhagic vascular complications.

The other patient died from renal failure six months after hospitalization.

No patient was admitted for acute coronary syndrome or needed consecutive coronary revascularization 3 years after the index procedure.

The patients had vascular complications such as limb ischemia in 2 cases and were treated by an antegrade perfusion, a femoral perforation in 1 case that needed surgical repair, and hematoma in 7 patients.

All the patients had a significant drop in hemoglobin of more than 2 g/dl and received blood transfusions.

Two patients were treated for septicemia, and 1 patient needed hemodialysis.

## 5. Discussion

We report our experience of using prophylactic ECMO during high-risk coronary angioplasty for inoperable patients.

The main findings of our study are as follows: (1) PCI can be successfully performed when adequate hemodynamic support is offered by the ECMO system. (2) Major adverse cardiac events such as cardiogenic shock or even cardiac arrest during high-risk PCI may be avoided by the use of prophylactic ECMO. (3) Despite the complexity and the potential risk of complications of the PCI procedure and the use of the ECMO system, patients who are good candidates for this type of mechanical revascularization seem to have a long-term clinical benefit at 3 years.

The main concern regarding the prophylactic use of ECMO is the selection of patients who really need mechanical circulatory support during coronary procedures for which conventional PCI could trigger hemodynamic instability with severe consequences.

For the moment, our community lacks a consensus on what defines high-risk PCI. Myat et al. suggested a definition based on clinical criteria (cardiogenic shock, severe left ventricular systolic dysfunction, Killip classes II–IV, etc.), anatomic criteria (the left main coronary artery, last remaining coronary conduit, SYNTAX score > 33, etc.), and hemodynamic criteria (the cardiac index <2.2 l/min/ml/m^2^, pulmonary capillary wedge pressure >15 mm Hg, and mean pulmonary artery pressure >50 mm Hg) [18].

The main reason to use ECMO during PCI in a prophylactic way is related to the high risk of hemodynamic instability due to prolonged ischemia after coronary manipulations in calcified coronary lesions, proximal segments, the need for aggressive devices like rotational atherectomy, or complex and long techniques like 2 stents bifurcation treatment or CTO.

Regarding the hemodynamic status, we can mainly encounter three different situations. The first one is instability despite pharmacological support. These patients seem to clearly benefit from prophylactic ECMO hemodynamic support before starting PCI [1].

The second situation concerns hemodynamically stable patients without recent heart failure episodes. Even if these patients are inoperable and are considered to be at high risks for PCI regarding anatomic and echocardiographic criteria, the decision to use ECMO before PCI is much more difficult to take because it is mainly based on the presumption of potential hemodynamic collapse during percutaneous revascularization. At present, this type of event is very difficult to predict, and we do not have accurate risk calculators to specifically assess the need for prophylactic ECMO in this particular setting [2].

The third category of patients is those who were stabilized under pharmacological support during acute myocardial infarction or acute heart failure in the context of severe coronary disease.

These patients are potentially at a higher risk of becoming unstable again during PCI, and therefore, prophylactic support by ECMO could be more advantageous in this specific scenario. Multiple parameters derived from right catheterisation and echocardiography were as follows: severe mitral regurgitation, severe aortic stenosis, depressed LV or RV function, severe pulmonary hypertension, high pulmonary capillary wedge pressure, and clinical signs of fluid overload could help the decision-making process about whether or not a specific patient requires hemodynamic support during high-risk PCI [3].

In our series, all patients had a recent acute heart failure episode related to myocardial infarction or progressive coronary disease. This aspect and also the probability of periprocedural prolonged disturbance of the coronary flow through the major epicardial artery were the main factors that were taken into account by our local heart team for the selection of patients that could benefit from ECMO-supported PCI. A complete analysis of potential risk complications related to VA-ECMO and PCI procedures was performed in all patients.

Local expertise with VA-ECMO and the availability of trained personnel are mandatory for this technique due to the high rate of complications. In our study, the major complications were vascular complications due to the size of the cannulas and also to the number of patients with peripheral arteriopathy. Patients with leg ischemia related to the occlusion of the femoral artery due to the arterial cannula underwent an antegrade perfusion of the leg (so-calledleg-ECMO or L ECMO), and one of the patients needed surgical repair for a femoral perforation.

A different prophylactic hemodynamic support using an Impella catheter that uses a smaller 14 Fr femoral sheath could be a good alternative in high-risk PCI cases [19].

Concerning the selection of the optimal mechanical hemodynamic support device, a series of parameters must be taken into account: the systemic flow needed, filling pressures, right ventricular function or pulmonary status, the specificity of the support technique, and most importantly, the local expertise. In the future, randomized trials will address these issues and establish the best algorithms to select the proper support device.

Our data confirm that the prophylactic use of VA-ECMO in a high-risk setting has a good long-term outcome even for extremely critical patients that would be treated otherwise conservatory and would probably have a poor short-term prognosis.

The particularity of our study is the long-term clinical follow-up of three years and the clinical characteristics of our patients who presented a recent acute heart failure episode, an important element that could be taken into account in the decision-making process of using or not prophylactic VA-ECMO.

The main limitations of our study are the observational nature, the single-center enrolment, and the limited sample.

## 6. Conclusions

Prophylactic use of ECMO in elective patients is an acceptable strategy for revascularization by high-risk coronary percutaneous intervention with good long-term outcomes for patients considered inoperable when a clear clinical benefit is expected. Regarding the potential risk of complications due to the VA-ECMO system, the selection of candidates in our series was based on a multiparameter analysis. The two main triggers in favor of prophylactic VA-ECMO in our studies were the presence of a recent heart failure episode and the high probability of periprocedural prolonged impairment of the coronary flow through the major epicardial artery.

## Figures and Tables

**Table 1:** Baseline clinical characteristics.

Patient	Age (years)	Males	BMI (kg/m^2^)	Risk factors	KD	ACS	Pulmonary edema at admission	Cardiogenic shock at admission	Congestive heart failure	Previous stroke
1	67	1	25.6	H HT T PAD	1	UA	1	0	0	0
2	76	1	27.1	HT T	0	NSTEMI	1	C	0	0
3	74	1	22.3	H HT T	1	NSTEMI	1	B	0	0
4	70	1	28	D H HT T PAD	1	0	0	0	1	0
5	74	1	27	H HT T PAD	0	NSTEMI	0	0	1	0
6	61	1	22.3	H HT T	1	NSTEMI	0	0	1	0
7	63	1	26.3	H HT T	1	0	0	0	1	0
8	74	1	22.4	H HT T	0	NSTEMI	0	A	1	0
9	66	1	26	H HT T PAD	0	0	1	0	1	0

BMI: body mass index; risk factors (diabetic (D) hypertension (H)/hypercholesterolaemia (HT)/tobacco abuse (T)/peripheral artery disease (PAD)); KD: kidney disease; impaired renal function (eGFR < 60, stage ≥ 3); ACS: acute coronary syndrome; UA: unstable angina; NSTEMI: non-ST-elevation myocardial infarction; cardiogenic shock stages: A, B, C, D, and E (SCAI definition).

**Table 2:** Baseline characteristics (cardiac history, anatomy, and risk scores).

Patient	Prior PCI	Prior CABG	Coronary artery	LVEF (%)	RV dysfunction	PH	EuroSCORE	EuroSCORE II (%)	SYNTAX 1	Residual SYNTAX
1	0	1	LM	30	1	1	18	41.94	38	10
2	0	0	LM	30	1	1	19	42.3	28	0
3	0	0	LM	50	1	1	10	6.51	26	0
4	1	0	LM	30	1	1	12	11.36	36	10
5	0	0	IVA	15	0	1	14	11.36	28	0
6	0	0	IVA, RCA	15	0	0I	15	14.78	26	0
7	1	0	Marginal circumflex/RCA	10	1	0	8	7.72	28	0
8	0	0	LAD	20	1	1	13	14.78	16	0
9	0	0	LM	10	0	0	10	16.35	38	0

PCI: percutaneous coronary intervention; CABG: coronary artery bypass graft surgery; coronary artery: LM (left main); LAD: left anterior descending; RCA: right coronary artery; CX: circumflex artery; LVEF: left ventricular ejection fraction. Right ventricular (RV) dysfunction was defined as a right ventricular ejection fraction ≤ 45% severe pulmonary hypertension (PH), defined by >50 mmHg after right catheterisation measurements.

**Table 3:** PCI characteristics.

Patient	Distal radial artery access	Right radial artery access	Guiding catheters	Multivessel PCI	Bifurcations with 2-stent technique types	Rotational atherectomy	IVL	Specific coronary balloons	Procedure duration	ECMO decannulation time (h)
1	0	1	7	0	1	0	0	1	1 h 43 min	5
2	1	0	7	0	1	0	0	1	1 h 5 min	–
3	0	1	7	0	1	0	0	1	1 h 22 min	20
4	0	1	7	0	1	0	0	1	1 h 15 min	6
5	0	1	7	0	1	0	0	1	2 h 20 min	6
6	1	0	7	1	0	1	0	1	1 h 49 min	20
7	0	1	7	0	1	0	0	0	2 h 12 min	6
8	0	0	7	1	1	1	1	1	2 h 23 min	48
9	0	1	7	1	1	1	0	1	1 h 48 min	6

IVL: intravascular lithoplasty; specific coronary balloons: cutting balloons, scoring balloons, and very high pressure balloons (OPN).

**Table 4:** Clinical outcomes.

Patient	PCI successful	Vascular complications	Need for hemodialysis	Hemoglobin drop (2 g/dl)	Septicemia	Total hospitalization length (days)	MACE at 30 days	MACE at 3 years
1	1	1	0	1	0	2	1	1
2	1	0	0	0	0	20	0	0
3	1	0	0	0	1	7	0	0
4	1	0	0	0	0	9	0	0
5	1	1	0	1	0	9	0	0
6	1	0	0	0	0	7	0	0
7	1	1	0	1	1	53	0	1
8	1	1	1	1	0	49	0	0
9	1	1	1	1	0	8	0	0

MACE: cardiac death: all-cause death; myocardial infarction; target vessel revascularization; stent thrombosis; stroke.

## Data Availability

The data can be made available from the corresponding author on request through an institutional review board.

## Abbreviations

PCI: Percutaneous coronary intervention
HR-PCI: High-risk PCI
LM: Left main
CTOs: Chronic total occlusions
CKD: Chronic kidney disease
COPD: Chronic obstructive pulmonary disease
HVD: Severe heart valvular disease
CABG: Coronary artery bypass graft
ACS: Acute coronary syndrome
PAD: Peripheral artery disease
TIA: And previous history of transient ischemic attack or stroke
VA-ECMO: Venoarterial extracorporeal membrane oxygenation
IABP: Intra-aortic balloon pump
eGFR: Estimated glomerular filtration rate
MI: Myocardial infarction
HF: Heart failure
NSTEMI: Non-ST-elevation myocardial infarction
TAP: The T and small protrusion technique.
